# Nanomaterial-Based Advanced Oxidation/Reduction Processes for the Degradation of PFAS

**DOI:** 10.3390/nano13101668

**Published:** 2023-05-18

**Authors:** Inês M. F. Cardoso, Luís Pinto da Silva, Joaquim C. G. Esteves da Silva

**Affiliations:** Chemistry Research Unit (CIQUP), Institute of Molecular Sciences (IMS), Department of Geosciences, Environment and Territorial Planning, Faculty of Sciences, University of Porto (FCUP), Rua do Campo Alegre s/n, 4169-007 Porto, Portugal; up201704720@edu.fc.up.pt (I.M.F.C.); luis.silva@fc.up.pt (L.P.d.S.)

**Keywords:** poly/perfluoroalkyl substances, advanced reductive processes, advanced oxidation processes, nanomaterials, water treatment

## Abstract

This review focuses on a critical analysis of nanocatalysts for advanced reductive processes (ARPs) and oxidation processes (AOPs) designed for the degradation of poly/perfluoroalkyl substances (PFAS) in water. Ozone, ultraviolet and photocatalyzed ARPs and/or AOPs are the basic treatment technologies. Besides the review of the nanomaterials with greater potential as catalysts for advanced processes of PFAS in water, the perspectives for their future development, considering sustainability, are discussed. Moreover, a brief analysis of the current state of the art of ARPs and AOPs for the treatment of PFAS in water is presented.

## 1. Introduction

Poly- and perfluoroalkyl substances (PFAS) are a huge class of mainly fluorinated anionic surfactants, consisting of a hydrophobic carbon–fluorine straight or branched chain and a hydrophilic functional group, with over 3000 compounds that have been produced and used since the 1950s [1]. PFAS can be classified under three subclasses [2]: perfluoroalkyl sulphonic acids (PFSA); perfluoroalkyl carboxylic acids (PFCA); and fluorotelomer-based substances (precursor compounds). The two most representative PFAS are PFCA perfluorooctanoic acid (PFOA) and PFSA perfluorooctane sulfonate (PFOS), because they have been extensively produced and studied [3]. Due to their persistence, bioaccumulation and toxicity, some of these substances are now listed in the Stockholm Convention as new POPs [4]. Human exposure to these substances mainly occurs through food ingestion and drinking water [3]. PFAS have been detected in the wastewaters of municipal wastewater treatment plants without known direct industrial sources [5]. 

Currently, due to a myriad of applications [1,2,3,4,5,6,7], PFAS are ubiquitous in environmental water, and sustainable remediation strategies must be designed to improve the quality of water and reduce the burden of human health risks. However, due to the high chemical strength of the carbon–fluorine bond, PFAS are known to be highly recalcitrant to conventional water treatment processes [2,6,7]. Due to this limitation, adsorption, membranes and ion-exchange technologies, which are efficient in their capture from water sources, have been proposed [8,9,10,11,12]. However, these technologies do not destroy PFAS and produce solid wastes that require incineration, which raises serious sustainability questions [6]. High-energy technologies, such as plasma, electron beams and gamma rays, have been successful in the destruction of PFAS, but their practical use is limited [2,6]. Moreover, some mechanochemical, electrochemical and sonochemical methodologies have shown potential, but major up-scaling challenges limit their utilization [2,6].

Advanced reduction and oxidation processes (ARPs and AOPs) using ozone, persulfate, hydrogen peroxide and ultraviolet can be easily implemented in water treatment stations, and they have shown promising results in the degradation of PFAS [13,14,15,16]. Persulfate-based AOPs that generate sulphate radical seem to be a better oxidant of PFAS [13,16]. Photochemical heterogeneous catalytic AOPs have been shown to degrade efficiently PFAS [5,6,7,17,18].

Nanomaterial-based treatment technologies for PFAS in water have been proposed taking into consideration their high surface-specific area (SSA), which would improve their adsorption capabilities and their designed increased reactivity, particularly as photocatalysts [19,20,21,22,23]. Titanium and indium oxides have been shown to be particularly active in the catalysis of the photochemical decomposition of PFAS [19,21,22]. Carbon-based nanomaterials, namely carbon nanotubes and graphene and its derivatives, have also been proposed for the degradation of PFAS, taking into consideration their high hydrophobicity, which is particularly adjustable for the adsorption of the hydrophobic carbon–fluorine chain of PFAS [23,24,25,26].

The objective of this review is to present and critically discuss the current standard ARPs and AOPs that can be used for PFAS degradation in water, and to review the nanomaterials that are currently being proposed to be coupled to advanced processes for water PFAS treatment. In the case of nanomaterials, this review will mainly focus on the publications and research trends in the last two years. With this review, we aim to rationalize the nanomaterials design to be coupled to AOPs/ARPs for PFAS mineralization, and to propose some directives for future research in this area.

## 2. Advanced Oxidation/Reduction Processes

AOPs and ARPs combine activation methods and chemical agents to form reactive radicals that degrade PFAS compounds. The degradation efficiency can be measured using different indicators, such as the “F index”, overall defluorination ratio (overall deF%) and molecular defluorination ratio (molecular deF%):

F index = [F^−^]_released_/[PFAS]_degraded_ (1)

Overall deF% = 100 × [F^−^]_released_/{[PFAS]_0_ × N_C-F_} (2)

Molecular deF% = 100 × [F^−^]_released_/{[PFAS]_degraded_ × N_C-F_} (3)

where [F^−^]_released_ is the molar concentration of fluoride released; [PFAS]_0_ is the initial molar concentration of PFAS; [F^−^]_degraded_ is the molar concentration of PFAS degraded; and N_C-F_ is the number of C-F chemical bounds in the parent PFAS molecule.

### 2.1. ARP

The molecular degradation processes in ARPs typically involve the hydrated electron (e_aq_^−^), hydrogen atoms (H*) and others, due to the specific chemical that is used; for example, when coupled with sulfite anions, sulfite radical anions (SO_3_*^−^) are also produced [27,28,29,30,31,32,33,34,35,36].

#### 2.1.1. Ultraviolet Light (254 nm) Systems

The photoionization of sulfite in water under standard UV light (254 nm) (UV systems) is described by the following chemical equation:

SO_3_^2−^ + *hν* 🡪 SO_3_*^−^ + e_aq_ (4)

The reactivity of the hydrated electron is capable of breaking the carbon–fluorine chemical bond of the PFAS, and in a treatment process its concentration must be maximized. pH is an important factor in hydrated electron speciation because it reacts with protons, according to the following equation:

e_aq_^−^ + H^+^ 🡪 H* (5)

However, if alkaline pH is used (for example, pH = 10–12), the hydrated electron is regenerated according to the following equation [37,38]:

H* + OH^−^ 🡪 H_2_O + e_aq_^−^ (6)

Therefore, alkaline conditions are preferred for the degradation of PFAS in water. The presence of the anions nitrate and carbonate quenches the hydrated electron, inhibiting its degradation efficiency [38]. Moreover, increasing the temperature and the solute dose increases the degradation and defluorination efficiencies—in the case of the solute dose, a critical value is observed above which no increase occurs [27].

**Figure 1:**
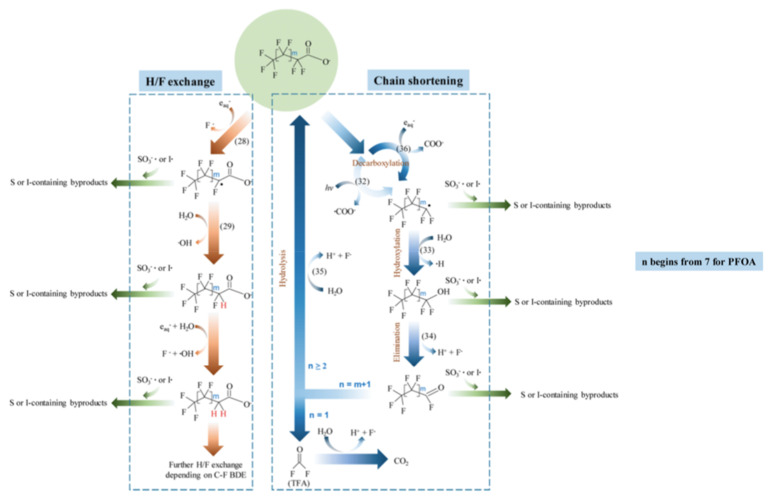
Proposed major pathways of reductive degradation of PFOA. Adapted with permission from reference [27]. Copyright {2020} American Chemical Society.

**Figure 2:**
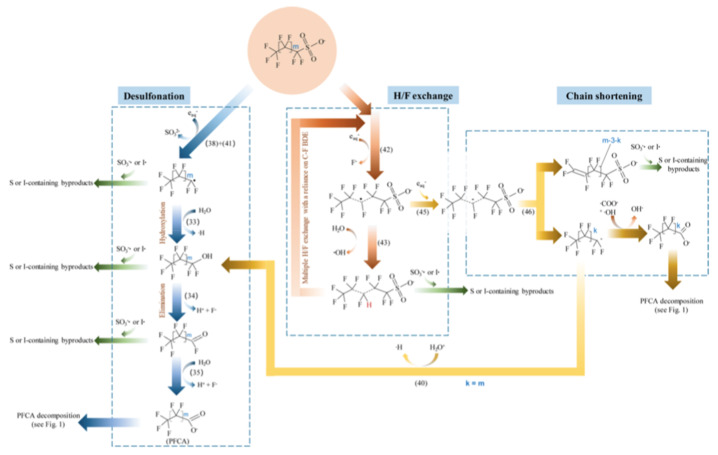
Proposed major pathways of reductive degradation of PFOS. Adapted with permission from reference [27]. Copyright {2020} American Chemical Society.

The degradation mechanism of the PFOA molecules by the hydrated electron is followed by H/F exchange and chain shortening (Figure 1) [27]. In the case of PFOS, besides similar H/F exchange and chain shortening via C-C cleavage, a desulfonation mechanism is observed (Figure 2) [27].

#### 2.1.2. Vacuum Ultraviolet Light (185 nm) Systems

Contrary to common UV systems, vacuum ultraviolet light systems (VUV) emitting in the high-energy wavelength region (185 nm/647 kJ/mol) can directly decompose PFAS molecules [39,40]. Moreover, these systems can produce more hydrated electrons due to water photolysis (*OH—hydroxyl radical):

H_2_O + *hν* 🡪 H* + *OH (7)

H_2_O + *hν* 🡪 H^+^ + e_aq_^−^ + *OH (8)

In order to improve hydrated electron production, VUV is coupled to the chemical agents iron(III) in acid aqueous solution [39] and sulfite in neutral-to-alkaline aqueous solutions [40]. In these cases, Equation (4) is also observed and, when iron(III) is present, the formation of complexes between ferric ions and PFOA improves the degradation efficiency [39].

When ferric ion is used in VUV, the defluorination rates increase 2.6 times [39]; when sulfide is present, the PFOS decomposition rate increases by nearly 7.5 and 2-fold faster than that in sole VUV and UV/sulfite systems, respectively [40].

In the case of VUV/sulfite, the degradation mechanism depends on the pH of the solution: at pH = 6 the direct photolysis of PFOS is observed; meanwhile, in alkaline condition (pH > 9), decomposition occurs via hydrated electron induction [40].

### 2.2. AOPs

Different activation methods are commonly used in AOPs that can easily be scaled up in real water or wastewater treatment stations; this review focuses on ozone, UV and heterogeneous photocatalysis.

The molecular degradation processes in AOPs are designed for the production of the hydroxyl radical (*OH), or other reactive oxygen species (ROS) that can be transformed into the hydroxyl radical, such as superoxide (O_2_*^−^) and peroxyl (HO_2_*) radicals. *OH radicals are extremely reactive oxidizers (the oxidation potential of the OH radical is approximately 2.8 V) and non-selective towards organic pollutants in water [41,42,43]. Other specific strong oxidant radical species can be produced when other chemical precursors are coupled with basic AOPs: for example, persulfate anion that generates the sulfate radical (SO_4_*^−^) [43].

The degradation of PFAS by the hydroxyl radical was denoted a minor pathway, as justified using experimental conditions where the production yield of this radical was low [44,45,46,47]. However, other different experimental conditions of the AOP resulted in quantitative degradation efficiencies, which makes ozone, and particularly when coupled with other agents, useful AOP for the degradation of PFAS. Some of the observed low efficiencies of AOPs in PFAS degradation are related to the high reduction potential of fluorine (Eº = 3.6 V), which makes the oxidation of fluoride to elemental fluorine thermodynamically unfavorable [46].

#### 2.2.1. Ozone

Bubbling ozone gas in water generates a mixture of unstable reactive species where hydroxyl radical is the major secondary oxidant [41,42]. The decomposition of ozone initiates with the reaction with hydroxide ions, involving reactions (9) to (11); depending on the pH of the aqueous solution, it evolves according to the different mechanisms depicted in reactions (12) to (16) [42].

O_3_ + OH^−^ 🡪 HO_2_^−^ + O_2_ (9)

O_3_ + HO_2_^−^ 🡪 *OH + O_2_*^−^ + O_2_ (10)

O_3_ + O_2_*^−^ 🡪 O_3_*^−^ + O_2_ (11)

pH <≈ 8

O_3_*^−^ + H^+^ ⇌ HO_3_* (12)

HO_3_*^−^ 🡪 *OH + O_2_ (13)

pH >≈ 8

O_3_*^−^ ⇌ O*^−^ + O_2_ (14)

O*^−^ + H_2_O 🡪 *OH + OH^−^ (15)

*OH + O_3_ 🡪 HO_2_* + O_2_ (16)

To increase hydroxyl radical production, ozone can be coupled to hydrogen peroxide (H_2_O_2_) in the so-called peroxone process [48,49]. The hydroxyl radical production mechanism in the peroxone process is [49]:

H_2_O_2_ ⇌ HO_2_^−^ + H^+^ (17)

HO_2_^−^ + O_3_ 🡪 HO_2_* + O_3_*^−^ (18)

O_3_*^−^ + H^+^ 🡪 HO_3_*^−^ (19)

HO_3_*^−^ 🡪 O_2_ + OH* (20)

OH* + H_2_O_2_ 🡪 HO_2_* + H_2_O (21)

The analysis of Equation (21) shows that the relative amount of hydrogen peroxide can provoke the quenching of the hydroxyl radical. Consequently, there exists an optimum ratio between ozone/hydrogen peroxide that maximizes the production of hydroxyl radical and the efficiency of the peroxone process [49].

Ozone can be coupled with 254 nm of UV light (ozone/UV process), which generates further hydroxyl radical, mainly according to the following chemical equations [49]:

O_3_ + *hν* 🡪 O_2_ + O* (22)

O* + H_2_O 🡪 2 *OH (23)

The catalytic degradation of ozone can also promote the formation of hydroxyl radicals enhancing the degradation efficiency of organic molecules. For example, the presence of iron(II) may result in the following chemical equations [50]:

Fe^2+^ + O_3_ 🡪 FeO^2+^ + O_2_ (24)

FeO^2+^ + H_2_O 🡪 Fe^3+^ + *OH + OH^−^ (25)

Molecular ozone has a redox potential of Eº = 2.07 V and *OH an Eº = 2.8 V, characteristics that are insufficient for the quantitative degradation of PFAS [46]. The degradation of PFSA and PFCA using 0.300 g of ozone per hour achieved about 22% degradation [46]. However, in another work, PFAS molecules were quantitatively degraded with ozone air fractionation [47]. Moreover, the degradation of PFOA and PFOS achieved 85 up to 100% via ozonation (2.5 wt% O_3_ generated at 85 W at 8.7 g of ozone per hour) under alkaline conditions (pH = 11) [44]. Contrary to these last studies, PFOA was only 0.5% defluorinated in 4 h of reaction time using ozone at 0.025 g per hour [14]. The analysis of these results with PFAS and ozone apparently shows that the higher the ozone dosage, the higher the degradation efficiency; moreover, alkaline pH values increase the degradation efficiency. Nevertheless, apparently the optimum ozone concentration has a threshold due to a self-quenching of the hydroxyl radical [51]. However, the effect of the hydroxyl radical in the PFAS degradation is controversial, and a study has shown that it is ineffective in that role [52].

Coupling ozone with other agents usually increases the PFAS degradation yield. Bubbling ozone in a photoreactor containing a 28 W mercury lamp (254 nm) and the photocatalyst TiO_2_ allowed a 4.18-times increase in defluorination when compared with the UV/O_3_ system [14]. Coupling ozone with H_2_O_2_ resulted in enhanced PFAS removal rates [44]. Using ozone/persulfate in pilot-scale experiments resulted in an overall 77% PFAS removal improvement and a degradation higher than 98% of the long-chain PFAS [46].

#### 2.2.2. UV Degradation Techniques

UV radiation is subdivided into four wavelength regions according to its energy: vacuum UV (VUV), 100–200 nm; UVC, 200–280 nm, which includes the standard 254 nm; UVB, 280–315 nm; and UVC, 315–400 nm. The energy that corresponds to UV ranges that are usually used in water treatment, VUV and UVC, is 646.8 kJ/mol (185 nm) and 471.1 kJ/mol (254 nm), respectively [53]. Moreover, C-C and C-F bond energies are 347 and 552 kJ/mol, respectively, meaning that PFAS should not be, at least, fully photolyzed by the 254 nm UV radiation, which is the most common used UV source because it is commercially available for water disinfection applications [54].

The direct degradation of PFOA by a 254 nm UV lamp is, as expected, limited [28,52,53,54,55,56]. Relatively high degradation rates (89% degradation and 33% defluorination yields) were only observed with a relatively high-power xenon–mercury lamp (200 W) and for a relatively high exposition time (72 h) [56]. The degradation under six UVC-254 nm 4 W lamps for 24 h resulted in 21% degradation and 9% defluorination yields [52].

UV AOP is commonly coupled to hydrogen peroxide to generate the hydroxyl radical (UV/H_2_O_2_), and the following mechanism is observed [43]:

H_2_O_2_ + *hν* 🡪 *OH (26)

H_2_O_2_ + *OH 🡪 HO_2_* + H_2_O (27)

H_2_O_2_ + HO_2_* 🡪 H_2_O + O_2_ + *OH (28)

*OH + *OH 🡪 H_2_O_2_ (29)

*OH + HO_2_* 🡪 H_2_O + O_2_ (30)

HO_2_* + HO_2_* 🡪 H_2_O_2_ + O_2_ (31)

However, as discussed in the previous ozone section, *OH generation methods are generally ineffective in the degradation of PFAS [53]. Consequently, useful UV PFAS degradation processes should be based on other reactive radical species besides the hydroxyl radical, for example in the system UV/persulfate anion (S_2_O_8_^2−^). Here, the following chain mechanism is obtained upon activation (heat, UV, iron, etc.), which generates the highly reactive sulfate radical (SO_4_*^−^) [13,43]:

S_2_O_8_^2−^ + *hν* 🡪 2 SO_4_*^−^ (32)

H_2_O + SO_4_*^−^ 🡪 HSO_4_^−^ + *OH (33)

S_2_O_8_^2−^ + SO_4_*^−^ 🡪 S_2_O_8_*^−^ + SO_4_^2−^ (34)

*OH + S_2_O_8_^2−^ 🡪 S_2_O_8_*^−^ + OH^−^ (35)

*OH + *OH 🡪 H_2_O_2_ (36)

SO_4_*^−^ + *OH 🡪 HSO_5_^−^ (37)

SO_4_*^−^ + SO_4_*^−^ 🡪 S_2_O_8_^2−^ (38)

**Figure 3:**
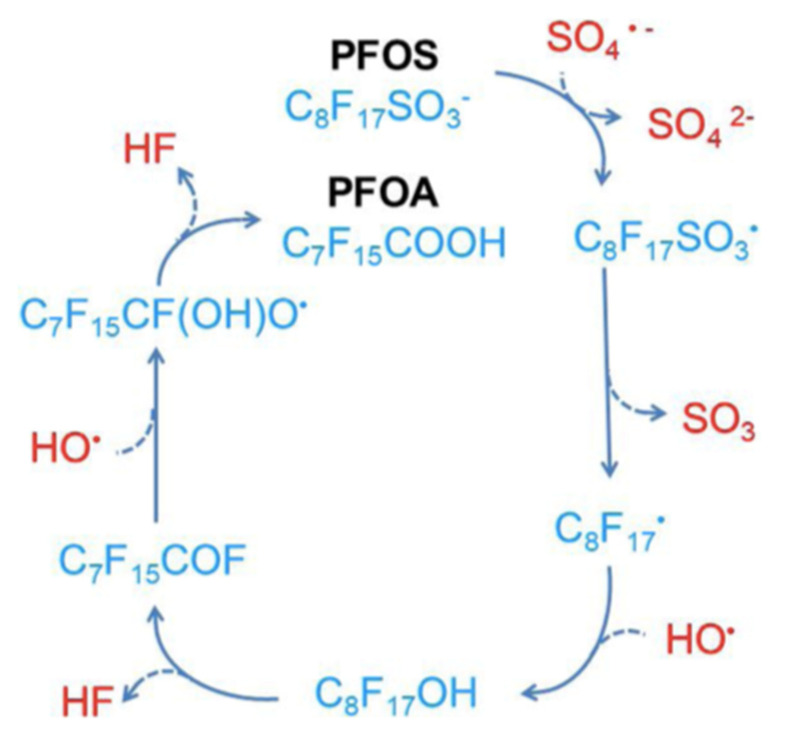
Proposed mechanisms for the conversion of PFOS to PFOA in the persulfate-based system. Adapted with permission from reference [13]. Copyright {2020} Elsevier B.V.

**Figure 4:**
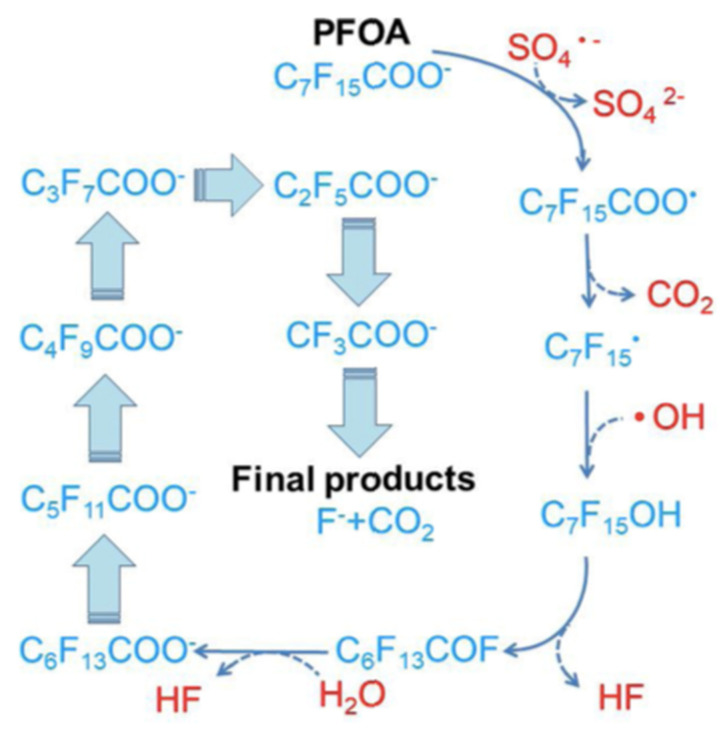
Proposed pathway for PFOA decomposition in the persulfate-based system. Adapted with permission from reference [13]. Copyright {2020} Elsevier B.V.

The sulfate radical has a higher oxidation potential (2.5~3.1 V) and a longer lifetime than the hydroxyl radical [13] and is demonstrated to be effective in PFOS and PFOA degradation [57,58,59,60,61]. Indeed, the activation of persulfate by UV-visible light from a xenon–mercury lamp (200 W—220 to 460 nm) in an acid solution (pH = 3.0–3.1) containing PFOA (1.35 mM) resulted in its complete degradation in 4 h, and its transformation into fluoride, CO_2_ and short-chained PFCA [57]. The activation of persulfate using hot water (60 to 80 °C) successfully degraded hydroperfluorocarboxylic acids (H-PFCAs) (371–392 microM), achieving 96.7 to 98.2% mineralization after 6 h [58]. A similar temperature (85 °C) activated persulfate procedure was used with PFOA (200 nanog/L) and, after 30 h, a 93.5% degradation with 43.6% of F^−^ yield was observed, together with the detection of shorter chain length compounds (C_6_F_13_COOH, C_5_F_11_COOH, C_4_F_9_COOH and C_3_F_7_COOH) [59]; this study also showed that lowering the pH and the temperature reduces the degradation efficiency. Several activators for persulfate were compared for the degradation of PFOS (0.186 mM), and the following order was observed: hydrothermal (22.52% defluorination efficiency, 12 h) > UV (254 nm) > Fe^2+^ > ultrasound [60]. Figure 3 and Figure 4 show a hypothesis for the mineralization mechanism of PFOS [13].

#### 2.2.3. Heterogeneous Photocatalysis

Using titanium dioxide (TiO_2_) as a typical heterogeneous photocatalyst and semiconductor, an AOP based on heterogeneous photocatalysis exposed to ultraviolet and/or visible light has the following mechanism of reactive substances production [43]:

TiO_2_ + *hν* 🡪 TiO_2_ (e_CB−_ + h_VB+_) (39)

TiO_2_(h_VB+_) + H_2_O 🡪 TiO_2_ + H^+^ + *OH (40)

TiO_2_(h_VB+_) + OH^−^ 🡪 TiO_2_ + *OH (41)

TiO_2_(e_CB−_) + O_2_ 🡪 TiO_2_ + O_2_*^−^ (42)

After TiO_2_ absorption, an electron (e_CB−_) in the valence band is transferred to the conduction band, leaving a hole in the valence band (h_VB_+). A typical example of the application of TiO_2_ for PFAS treatment is when an iron halogenide UV lamp (500 W, 95 W/m^2^, 315–400 nm) irradiated a suspension of a commercial TiO_2_ sample, at a concentration of 0.66 g/L, degrading 4 mM of PFOA following pseudo-first order kinetics in the first 4 h, with an apparent rate constant of 0.1296 h^−1^ [62]. This study also detected shorter perfluorinated carboxylic acids, C_n_F_2n+1_COOH (n = 1–6) as intermediates and fluoride anion, which reacted with the surface of the photocatalyst and affected its efficiency.

Doping TiO_2_ with metal ions improves the photocatalytic efficiency of the semiconductor by producing traps in the molecular orbitals that capture the electrons and holes, reducing the electron–hole recombination, making them more available to react with organic molecules leading to their degradation [63]. Upon exposure to UV light (245 nm, 200 W), copper-doped TiO_2_ catalyzed the decomposition of PFOA after 12 h with an apparent rate constant of 0.186 h^−1^ and a defluorination rate constant of 0.462 mg/L h^−1^ [63]. The decomposition of the PFOA into fluoride ions and shorter PFCA, such as C_6_F_13_COOH, C_5_F_11_COOH, C_4_F_9_COOH, C_3_F_7_COOH, C_2_F_5_COOH and CF_3_COOH, was observed.

A material produced from low-cost commercial activated carbon and TiO_2_ was prepared, named Fe/TNTs@AC, including both synergistic adsorption and photocatalytic capabilities [64]. This material (in a concentration of 4 g/L), upon exposure for 4 h under UV irradiation (254 nm, 21 mW cm^–2^), was able to degrade more than 90% of PFOA with a 62% mineralization to fluoride ions in the pH range between 4 and 8. Using selective scavengers, it was concluded that the hole h_VB_+ is the important player in PFOA degradation—*OH and O_2_*^−^ did not play a particularly active role [64].

New material semiconductors with improved photocatalytic efficiencies, particularly in the visible wavelength range, have been prepared based on bismuth [17,65,66]. Based on the so-called “concentrate and destroy” strategy, a bismuth phosphate was coupled to carbon spheres (CS), resulting in the composite BiOHP/CS [17]. This composite (1 g/L) was able to quantitatively adsorb PFOA (initial concentration of 200 microg/L at pH = 7) in 2 h and achieve its almost complete decomposition in 4 h when irradiated by a UV light (18 W low-pressure Hg lamp, 254 nm, 21 mW/cm^2^).

**Figure 5:**
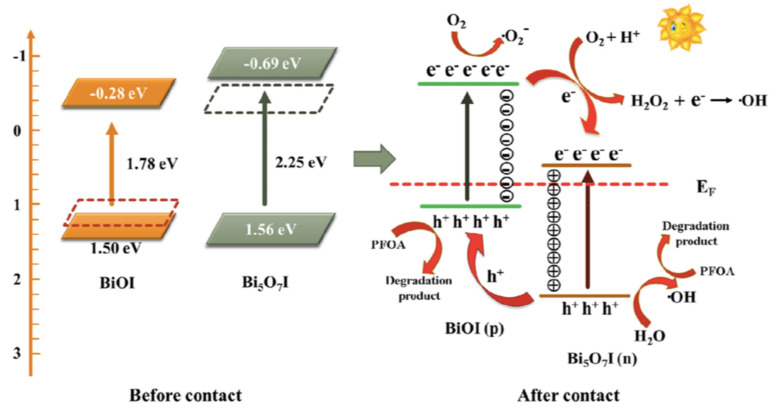
Proposed photocatalytic mechanism of PFOA in BiOI@Bi_5_O_7_I *p-n* heterojunction photocatalytic system. Adapted with permission from reference [65]. Copyright {2019} Elsevier B.V.

Bismuth oxohalides (BiOX, X - F, Cl, Br and I) are typical photocatalysts. BiOI has a narrow bandgap (e.g., 1.7–1.9 eV), enabling its use in the visible wavelength range, and several composites with it have been proposed for the treatment of PFAS in water [65,66]. The photocatalyst BiOI@Bi_5_O_7_I (0.5 g/L) degrades PFOA (15 mg/L) under simulated solar light with a rate constant of 0.247 h^−1^ (it removes about 60% of the organic carbon under 6 h of irradiation) [65]. The role of the reactive species *OH, O_2_*^−^ and h_VB_+ in PFOA decomposition was assessed using selective scavengers, and all the species were active in that mechanism. This study also showed that PFOA was decomposed by stepwise losing CF_2_ units (Figure 5). Bi_5_O_7_I/ZnO microspheres were used in PFOA degradation analysis when irradiated with simulated visible light (400 W Xe lamp with a 420 nm cutoff filter), and a degradation rate constant of 0.013 h^−1^ was obtained [66]. This work demonstrated that the degradation of the PFOA was initiated by the most reactive carboxylic acid group of PFOA, which was vulnerable to attack by the h_VB_+, followed by the successive elimination of CF_2_ units.

Taking into consideration that the potential quantity of water to be treated for PFAS is enormous, photocatalyst design must consider environmental and social/economical sustainability issues. A low-cost and sustainable zero-valence iron (PVP/Fe^0^) was used as a photocatalyst for the degradation under UV radiation (14 UVC light bulbs, 8 W each, centered at 254 nm, 4.24 mW/cm^2^) of a mixture of PFOA, PFOS and perfluorononanoic acid (PFNA) in real wastewater from WWTP [18]. The degradation percentages were (0.5 microg/L of each PFAS at pH = 3) 90, 88 and 43% for PFAN, PFOS and PFOA, respectively.

### 2.3. ARP vs. AOP

Table 1 displays information about the most PFAS-efficient AOP degradation methodologies that allow the comparison of the degradation procedures of PFAS. Taking into consideration the previous discussion and Table 1 data, we can conclude that the hydrated electron-based methodologies (ARP) and UV-generated sulfate radical (AOP) are more efficient for the PFAS degradation than the hydroxyl radical-based technologies and the photocatalytic methods.

## 3. Nanomaterials-Based AOPs and ARPs

**Figure 6:**
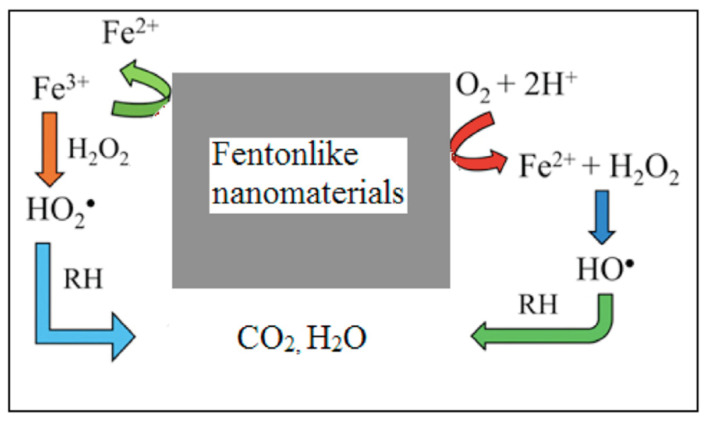
Schematic representation of the action of Fenton-like nanomaterials in the degradation of organics. Fe^2+^/Fe^3+^ redox couple generates the reactive free radicals involved in the degradation of toxic pollutants. Adapted with permission from reference [67]. Copyright {2021} Elsevier B.V.

The incorporation of nanomaterials in nearly all AOPs has already been made with promising results [67]. Due to its high SSA and specific designed surface, nanomaterials act as AOP catalysts, resulting in an increased production of reactive species under mild experimental conditions, improving degradation yields for lower contact times. Typical examples of these catalysts are those used in heterogeneous Fenton-like processes for the production of hydroxyl radical from hydrogen peroxide (Figure 6), mainly using iron-based nanomaterials [67].

Although with some identified limitations, as previously discussed, ARPs and AOPs show great potential for the treatment of water, either for human consumption and wastewater, contaminated with PFAS. However, the performance and the sustainability of those processes must be improved, because fast and high degradation yield processes must be developed, taking into consideration sustainability issues such as raw materials extraction and environmental impacts. Considering the increased reactivity, markedly lower mass content and large SSA, nanomaterials can definitely contribute to this optimization evolution of the PFAS treatment classical technologies.

### 3.1. Previous Reviews

Three previous reviews have been published about the use of nanomaterials in PFAS treatment technologies, discussing publications until the year 2021 [19,21,22]. The general analysis of these reviews shows that nanomaterials have been proposed with two main functions in the treatment technologies: general adsorption (the removal of PFAS from the water and its concentration in the nanomaterial) and the heterogeneous photocatalysis degradation of PFAS AOP.

The majority of the proposed nanomaterials used for adsorption water treatment technologies use carbon nanotubes (CNT) [68]. The use of carbon-based nanomaterials results from the good performance of the well-known granular activated charcoal (GAC), whose implementation is widespread for the adsorption of micropollutants, including PFAS, in classical water treatment technologies [69]. Moreover, CNTs have been proposed as highly promising materials for water treatment technologies [68], but their toxicity and potential health risks may compromise this application [70], making it imperative to research alternatives.

A carbon-based nanomaterial with much better sustainability characteristics than CNT is carbon dots (CD) [71,72]. CDs can be easily prepared from any carbon precursor, including carbon residuals [72], and have been proposed as sustainable nanomaterials [73,74]. However, CDs have only been described in a small number of papers as adsorbent materials for PFAS [75,76] and/or as sensors [77,78,79] for PFAS. Moreover, taking into consideration their versatility and straightforward functional design, these carbon-based nanomaterials show great potential to be used in PFAS AOP/ARP technologies.

Metal oxides nanoparticles are also used as nanoadsorbent platforms for PFAS because they have high SSA and many surface functional groups [22]. Since PFAS soluble in water have a negative charge, the adsorption is maximized when the pH is lower than the point of zero-charge (PZC) of the oxide, namely: Al_2_O_3_, 7.3; Fe_2_O_3_, 7.6; and TiO_2_, 5.4 [80]. Aside from the pH, the SSA and the surface hydroxyl density (SHD) are critical factors in the PFAS adsorption efficiency of the metal oxides, and the SSA (m^2^/g) and SHD (micromol/m^2^) are, respectively: Al_2_O_3_, 198 and 31.2; Fe_2_O_3_, 41.7 and 21.0; TiO_2_, 64.1 and 35.5; and SiO_2_, 278 and 18.3 [80]. Moreover, the formation of inner-sphere complexes at the surface of the metal ions by metal cations increases the adsorption capacity [22,80]. Other experimental factors contribute to the adsorption inhibition, namely the presence of negatively charged polymers, such as dissolved organic matter, which compete with PFAS for the adsorption sites and the agglomeration of the adsorbent nanoparticles. Nano alumina, hematite and goethite show better adsorption characteristics.

Semiconductor nanometal oxides, such as TiO_2_ (band gap—3.0–3.2 eV, ~400 nm), In_2_O_3_ (band gap—~2.9 eV, ~428 nm) and Ga_2_O_3_ (band gap—4.8 eV, 258 nm) have been proposed as photocatalysts in UV AOP [19,21]. Indeed, some of these nanomaterials correspond to the size reduction towards the nanometer dimensions of some of the bulk photocatalysts that have been used in classical UV AOP, as previously discussed in Section 2.2.3. Among the semiconductor nanometal oxides, In_2_O_3_ photocatalysts have been shown to have the highest potential for PFAS degradation due to its SSA and the type and amounts of reactive species that are generated upon UV irradiation [19]. Photocatalytic degradation efficiencies can be improved by doping metal oxides with CeO_2_ and noble metals (Ag, Pt, Pd) [22].

However, due to the costs and limited environmental resources, the use in large-scale plants of the metals described in the previous paragraph raises severe sustainability concerns. As an alternative, cheaper and more abundant metals are being used for PFAS degradation, for example Zn (mainly as ZnO), Fe (mainly as Fe^0^) and Mn (mainly as Mn_2_O_3_) [22]. Significant PFAS degradation is observed when these nanometals/nanometal oxides are coupled with UV/ozone or hydrogen peroxide, either under UV or visible radiation [22].

The most important characteristic of nanomaterials that makes them suitable to be coupled to ARPs/AOPs is their versatility. They can be designed to have a particular functionality or several functionalities, with a residual mass of resources, when compared with bulk materials. Moreover, taking into consideration their extraordinary high SSA, this results in higher reactivity.

Although the research on nanomaterials applications in the treatment of PFAS in water is independently oriented into two main lines, adsorption and degradation, the next step is the combination of these two functionalities in only one nanoparticle. Indeed, the strategy of “concentrate and destroy”, discussed above in Section 2.2.3, is the next step behind nanomaterials design for ARPs/AOPs for PFAS treatment.

### 3.2. Advances in Concentration Strategies of PFAS

**Figure 7:**
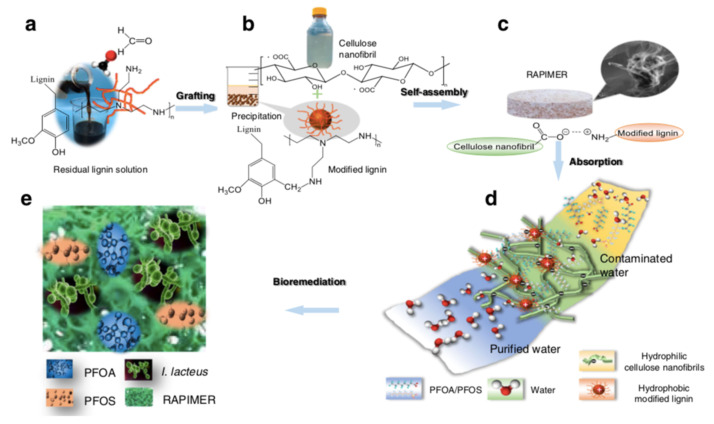
The design strategy, fabrication process, chemical adsorption and fungus degradation scheme of the RAPIMER system. (**a**) Corn stover residual lignin solution and selective graft reaction using formaldehyde and polyethylenimine to produce positively charged modified lignin particles. (**b**) Corn stover-derived cellulose nanofibrils prepared by TEMPO-oxidation method and modified lignin chemical structure. (**c**) The modified lignin and nanocellulose nanofibrils formed RAPIMER composite through self-assembly by the formation of carboxylic acid/amine interaction. (**d**) PFAS adsorption by the RAPIMER composite. (**e**) Fungal bioremediation through co-metabolism and biodegradation of PFAS and RAPIMER system. Adapted from reference [80].

Environmental sustainability concerns were translated into the proposal of technologies based on biological systems and nanomaterials [80,81,82]. A Renewable Artificial Plant for in situ Microbial Environmental Remediation (RAPIMER) was developed from chemically modified lignocellulosic biomass (Figure 7) [80]. RAPIMER is a nanomaterial, based on cellulose and lignin, that enables an efficient PFAS adsorption, provides a support for fungus and bacteria that will decompose PFAS, and supports the expression of redox enzymes to degrade PFAS. RAPIMER has a finer nanometric (2.35 nm) fiber structure, which results in a high SSA, and the negatively charged cellulose nanofibrils (hydrophilic) and the positively charged lignin (hydrophobic) generate a 3D amphiphilic environment, allowing PFAS strong adsorption due to charge attraction and hydrophobic interaction. RAPIMER has a PZC of 8.22 and adsorption decreases for pH below 8. Low concentrations of PFOA and PFOS (1 microg/L) in complex solutions were removed by RAPIMER at efficiencies of 99% or higher. The adsorbed PFAS inside RAPIMER were subjected to bioremediation.

PFAS suffered bioremediation in anaerobic reactor where carbon materials, including CNT, were supplemented as electron drivers [81]. Biological methods for PFAS environmental removal are under investigation [82] and are characterized as cost-effective, eco-friendly and involving simple operation. Nanomaterials (nanobiochar, CNT, nanometal oxides) are included in biological technologies as adsorbent nanoplatforms.

Foam fractionation technologies (FFT) have been proposed for the pre-treatment of water to remove soluble PFAS, as well as for producing low-volume high concentrated solutions for subsequent destruction [83,84,85]. Long-chain PFAS are usually removed with high efficiencies (>90%), while short-chain PFAS are removed with low efficiencies (<30%) [83]. Although nanomaterials have not been included in the FFM formulations for PFAS concentration, these technologies are also used for nanomaterials (silica nanoparticles and CNT) removal from wastewater [86]. Taking into consideration the active role of nanomaterials in the adsorption/degradation of PFAS based in several AOPs, the coupling of FFT for PFAS treatment with designed nanomaterials is an open window of research.

CNTs, both single-walled (SWCNT) and multi-walled (MWCNT), continue to be proposed as adsorbent platforms for PFAS, but SWCNTs show better adsorption performances due to the lower SSA of MWCNTs [87]. Moreover, the modification of CNTs with nano-MgAl_2_O_4_ has been proposed as an improved adsorbent for PFAS (100 ppb), allowing 99% removal after 3 h and 100% in 3.5 h [88]. The size of the nanocomposite MgAl_2_O_4_@CNT was 80 to 120 nm, with a SSA of 149.41 m^3^/g, a pore volume of 0.27 cm^3^/g and a pore size of 9.69 nm. The nanocomposite adsorbs PFAS by hydrophobic and electrostatic interactions and can be used at mild alkaline solutions.

Metal–organic frameworks (MOF), which are innovative nanopores ordered materials with high SSA and pore volumes, have shown an increased application for PFAS adsorption in recent years [87,89,90].

### 3.3. Advances in PFAS Degradation Technologies

**Figure 8:**
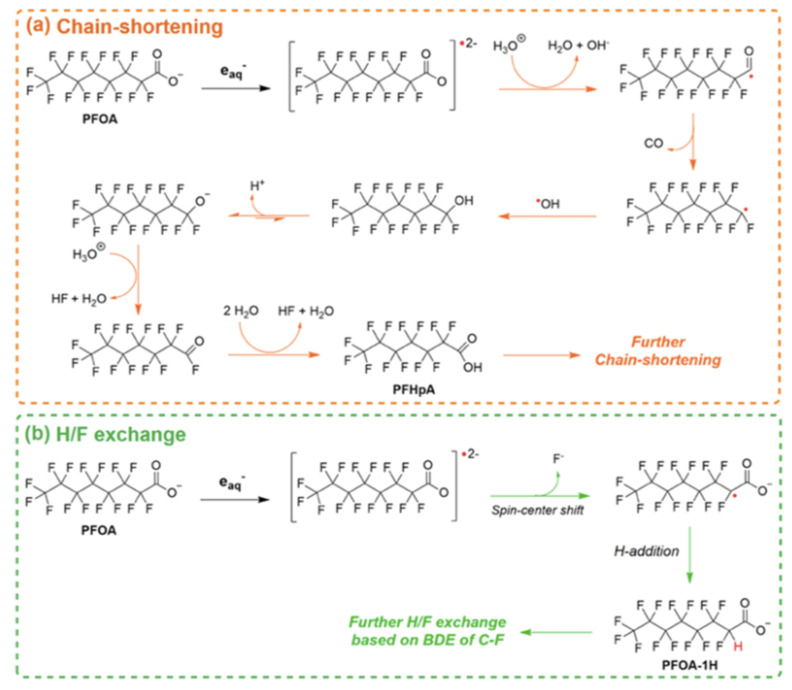
Plausible reaction mechanisms of (**a**) chain-shortening and (**b**) H/F exchange in the degradation of PFOA in the MIL-125-NH_2_ MOF system. Adapted with permission from reference [90]. Copyright {2022} American Chemical Society.

Besides their potential as adsorptive materials for PFAS, MOF, and particularly the titanium-based MIL-125-NH_2_, were used as photocatalysts for the degradation of PFOA under a 450 W mercury lamp [90]. After 24 h of irradiation, 98.9% degradation and a 66.7% defluorination rate of PFOA were obtained. Glucose, which is a critical factor for degradation, was used as non-hazardous sacrificial reductant, where it acted as a h_VB_^+^ scavenger. The degradation mechanism involves e_aq_^−^ and oxidizing reactive species (Figure 8).

Two-dimensional nanomaterials, Pt/La_2_Ti_2_O_7_ nanoplates and BiOF nanosheets, were prepared for use as photocatalysts of PFOA degradation [91,92]. La_2_Ti_2_O_7_ has a layered perovskite structure and is known to decompose water into hydrogen and oxygen by photocatalytic reduction under UV irradiation [91]. Pt was dispersed on the photocatalyst to improve catalytic activity. Irradiating a PFOA water solution without oxygen (bubbling nitrogen) using a UV light (254 nm, 1 mW cm^−2^), in the presence of Pt/La_2_Ti_2_O_7_ and methanol, as an electron donor, a 40% degradation was observed after 180 min and 50% degradation after 12 h. BiOF nanosheet photocatalysts were prepared with different amounts of ethylene glycol, taking into consideration that surface defects and/or exposed reactive facets should improve the photocatalytic performance [92]. The sample 50%-EG BiOF, under UV light, catalyzes the almost complete removal of PFOA and 56.8% removal of TOC.

**Figure 9:**
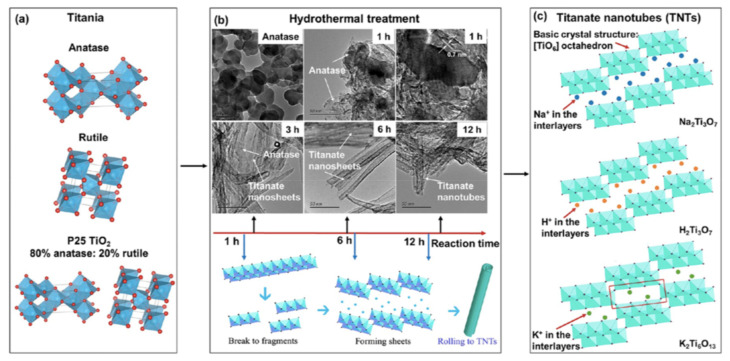
(**a**) Crystalline structures of TiO_2_ (red balls: O; dusty blue balls: Ti); (**b**) TEM morphologies of the original anatase and intermediate materials with different hydrothermal reaction times of 1−12 h, and schematic diagram of TNTs formation; (**c**) crystalline structures of typical TNTs (purple balls: O; blue balls: Na; orange balls: H; green balls: K). Adapted with permission from reference [93]. Copyright {2022} American Chemical Society.

One-dimensional titanate nanotubes (TNT) are TiO_2_ derivatives that have a uniform crystalline and scrolled tubular structure [TiO_6_], large SSA and high pore volume, good ion exchange ability, high photoelectric conversion properties and, for some derivatives, a high visible light response [93]. Figure 9 shows a scheme of the formation of TNT. Upon the solar irradiation of TNT, a similar mechanism of reactive substances production to TiO_2_ irradiated with UV, Equations (39)–(42), is observed. However, raw TNT cannot be used for PFAS adsorption (and eventually destruction) because the negative and hydrophilic surface of TNT repels the negative water-soluble PFAS. This limitation was overcome by doping TNT with photoactive metal oxides and by using activated charcoal (AC) as support (Metal/TNT@AC) [64,94,95,96]. These modifications allowed the development of PFAS “concentrate and destroy” technologies [64,94,95,96].

Ga/TNT@AC [94,95] and Bi/TNT@AC [96] were prepared and used for the adsorption and destruction of PFAS. The UV irradiation (210 W/m^2^) of Ga/TNT@AC (0.12 g) allowed for 75% degradation and 66.2% mineralization of PFOS (100 microg/L, pH = 7) within 4 h [94,95]. The photoactivity of Ga/TNT@AC was attributed to oxygen vacancies, which suppresses recombination and facilitates superoxide radical. Both the hole h_VB_+ and O_2_*^−^ played an important role in PFOS degradation. The UV irradiation (210 W/m^2^) of Bi/TNT@AC (1 g/L) allowed for 70% degradation and 42.7% mineralization of GenX (100 microg/L, pH = 7) within 4 h [96] (GenX is the ammonium salt of hexafluoropropylene oxide dimer acid and has been used as a PFOA replacement). The photoactivity of Bi/TNT@AC was attributed to hydroxyl radical and the hole h_VB_+.

Iron-based nanocomposites were proposed as adsorbents/catalysts for PFAS removal and degradation [18,20,97]. The removal of PFAS in wastewater effluents was successful using zero-valence iron nanoparticles coupled to UV light [18]. The degradation of PFAS in wastewater effluents was lower than in deionized water and higher at acid pH values (pH = 3); after 2 h, degradation rates of 90%, 88% and 46% were obtained for PFNA, PFOS and PFOA. Ferric hydroxide nanoparticles were synthesized in situ using ozone and the nanoparticles used for PFAS removal [20]. Although no PFAS destruction analysis was carried out, the adsorbent capacity of these nanoparticles was higher than conventional adsorbents, which, taking into consideration iron reactivity, have potential for the development of a “concentrate and destroy” process. An iron-clay(montmorillonite)-cyclodextrin(β-CD)-DFB (decafluorobiphenyl) was synthesized, with the iron–clay segment containing a heterogeneous Fenton catalyst function, while the CD-DFB was used as surface confinement for PFAS molecules [97]. This composite adsorbed >90% and oxidized >70% of long-chain PFAS and showed worse performance for short-chain PFAS. In the case of PFOA and PFOS, a 65% degradation was observed within 10 min.

New applications of TiO_2_ in novel photocatalysts have been investigated, such as the composite resulting from the calcination of boron nitrite (BN) with TiO_2_, BN/TiO_2_ [98]. The BN/TiO_2_ composite is more photoactive than the two precursors under UV light for PFOA, degrading 15 times faster than TiO_2_, with the active reactive species being photogenerated holes. Moreover, the lifetime of PFOA in outdoor experiments under natural sunlight, and in deionized water, was 1.7 h.

A UV Fenton reaction catalyzed by Fe_3_O_4_ nanoparticles was proposed for PFAS destruction [99]. The Fenton AOP involves the oxidation of a ferrous ion (Fe^2+^) by hydrogen peroxide in the presence of UV radiation, in order to promote the formation of reactive oxygen species, *OH and HO_2_* radicals [99]:

Fe^2+^ + H_2_O_2_ 🡪 Fe^3+^ + *OH + OH^−^ (43)

Fe^3+^ + H_2_O_2_ 🡪 Fe^2+^ + HO_2_* + H^+^ (44)

2H_2_O_2_ 🡪 *OH + HO_2_* + H_2_O (45)

**Figure 10:**
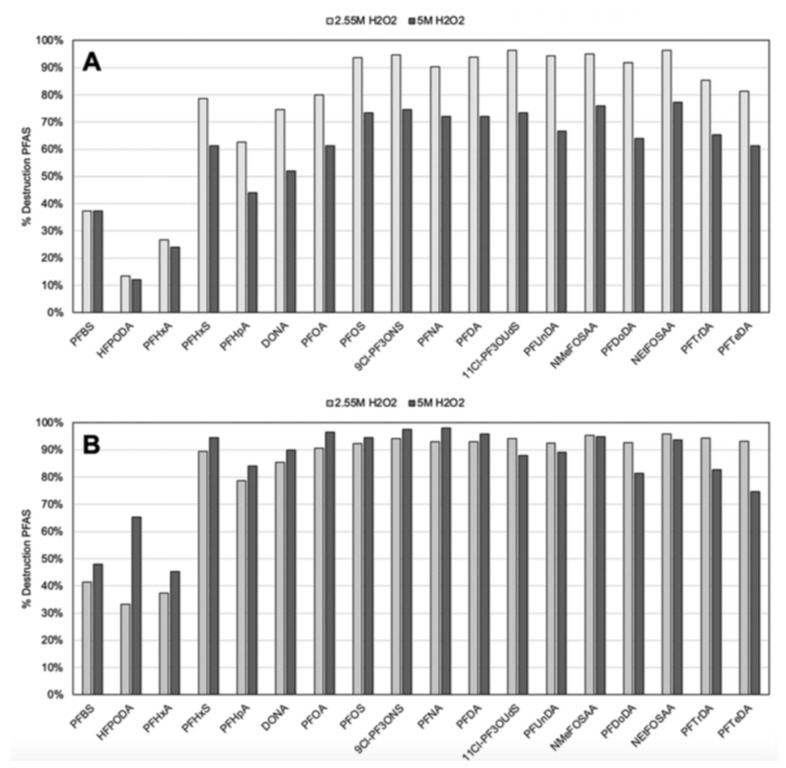
Percent destruction of 18 PFAS species at (**A**) pH 7 and (**B**) pH 9, and two concentrations of H_2_O_2_. All samples were prepared with 1000 ppm of Fe_3_O_4_ and underwent 30 min of UV-C exposure. Instrumental analysis measurement uncertainties for detected concentration (in units of ppt) are as follows: PFBS—23.2%; HFPODA *—28.3%; PFHxA—21.8%; PFHxS—21.6%; PFHpA—23.2%; DONA *—22.9%; PFOA—22.5%; PFFOS—25.2%; 9Cl-PF3ONS—22.3%; PFNA—21.5%; PFDA—23.2%; 11Cl-PF3OUdS *—25.9%; PFUnDA—22.8%; NMeFOSAA—27.1%; PFDoDA—24.4%; NEtFOSAA *—25.8%; PFTrDA—26.5%; PFTeDA—21.6%. * These PFAS species are branched, which may impact their reactivity when compared with the linear compounds. Adapted with permission from reference [99]. Copyright {2022} The Royal Society of Chemistry.

The UV Fenton system used six 15 W UV-C bulbs (wavelength 254 nm, 120 μJ cm^−2^), nano-magnetite (20–30 nm) and different pH values and hydrogen peroxide concentrations [99]. The samples were irradiated from 5 min to one-hour periods and left reacting for 24 h before analysis. Figure 10 shows the observed degradation efficiencies of the tested PFAS under this system. Approximately 90% degradation rates are observed, except for the short-chain PFAS, where much lower degradation percentages occur. Both nano-magnetite and H_2_O_2_ contribute to PFAS destruction, suggesting that ROS and the adsorption of PFAS in the surface of magnetite contribute to their destruction mechanism.

Table 2 presents the most important properties of the most recent nanomaterial PFAS degradation applications. As discussed previously, and from the observation of Table 2, we concluded that most of the degradation technologies are based on photocatalytic heterogeneous AOPs. The exception is a Fenton-like reaction technology based on the iron–clay–cyclodextrin composite [97]. Some photonanocatalysts have been shown to have high potential because they allow almost complete PFAS decomposition in a relatively short time—namely MOFs, such as nanomaterials, bismuth-based nanosheets and boron nitrite/titanium composites.

## 4. Perspectives

This review showed that classical advanced oxidation and/or reduction processes for water treatment can be used for the degradation of PFAS. Moreover, the advantages of coupling those processes with specific designed nanomaterials following the “concentrate and destroy” strategy was discussed. However, research on the coupling of nanomaterials and AOPs/ARPs is still highly insufficient because results are only available for heterogeneous catalyst AOPs.

Among ARPs/AOPs, those involving the hydrated electron and sulfate radical show higher PFAS degradation efficiency, particularly when UV light is used. Nanomaterials designed to be reagents and/or catalysts in these processes represent an open research field. Indeed, increasing PFAS degradation efficiency and reducing the UV irradiation is highly desirable. Moreover, extending the range of active wavelengths to the visible region is essential to minimize the economic and environmental costs of these processes. Alternatively, the use of sustainable nanomaterials could be used for the activation of reactive species (hydrated electrons and sulfate radicals) in the presence of sulfite or persulfate.

Indeed, the next designed strategy for PFAS nanomaterials/AOP for water treatment must be a “super-concentration/high-yield-destruction/sustainable” approach. This can be achieved using porous carbon-based nanomaterials, synthesized from biogenic waste, as the basis for a nanocomposite containing active dopants that will catalyze the overproduction of reactive molecular species in an ARP/AOP.

The research on new advanced process technologies will open new treatment windows for PFAS in water and for the development of new nanomaterials. For example, recently [100], it was observed that chlorine radicals (Cl* and Cl_2_*^−^) play an important role in the degradation of PFOA. Chlorine radicals are generated by UV/chlorine technologies. Moreover, it was discovered that PFCA can be mineralized under low-temperature conditions in the presence of hydroxide anion [101]. Indeed, the decarboxylation of PFCA occurs in polar non-protic solvent when the hydroxide anion is present, resulting in the production of a carbanion that undergoes decomposition. Nanoparticle design for non-aqueous solvents is still a challenge, but encouraging perspectives have been opened with this last discovery.

However, taking into consideration the large volumes of contaminated water that need to be treated, any viable technology needs to use economic and sustainable nanomaterials, for example, carbon-based carbon dots.

## Figures and Tables

**Table 1:** ARP/AOP selected processes for PFAS degradation.

PFAS	Process/Material	Main Reactive Species	%Deg	Time	Reference
PFOA (38.7 μM)	UV/sulfite (pH = 9.2)	e_aq_^−^	100%	10 min (200–400 nm, 250 W)	[37]
PFOA (10 μg/L)	UV/sulfite (pH = 12)	e_aq_^−^	100%	1 h (254 nm, 11 W)	[38]
PFOA (1.35 mM)	UV/Persulfate	SO_4_*^−^	100%	4 h (Xenon, 200 W)	[57]
PFOA (0.5 μM)	85 °C/Persulfate	SO_4_*^−^	93.5%	30 h	[59]
PFOA (50 mg/L)	UV/Cu–TiO_2_ (pH = 5)	*OH	91%	12 h (254 nm, 400 W)	[64]
PFOA	Fe/TNTs@AC (pH = 7)	h_VB_+	91.3%	4 h (254 nm, 21 mW cm^–2^)	[64]
PFOA (15 mg/L)	BiOI@Bi_5_O_7_I	*OH, O_2_*^−^, h_VB_+	60%	6 h (Xenon, 800 W)	[65]

**Table 2:** ARP/AOP coupled to nanomaterials for PFAS degradation.

PFAS	Material	Main Reactive Species	%Deg	Time	Reference
PFOA (100 μg/L)	MOF (MIL-125-NH_2_/Glucose)	e_aq_^−^, *OH	98.9%	24 h (254 nm, 450 W)	[90]
PFOA (50 ppb)	Pt/La_2_Ti_2_O_7_ nanoplates (Methanol)	e_aq_^−^	40%	180 min (254 nm, 1 mW∙cm^– 2^)	[91]
PFOA (15 μg/L)	BiOF nanosheets	O_2_*^−^, h_VB_+	100%	6 h (254 nm, 11 W)	[92]
PFOS (100 μg/L)	Ga/TNT@AC	O_2_*^−^, h_VB_+	75%	4 h (UV, 210 W/m^2^)	[94,95]
Gen-X (100 μg/L)	Bi/TNT@AC	*OH, h_VB_+	70%	4 h (UV, 210 W/m^2^)	[96]
PFOA/PFOS	iron-clay-cyclodextrin composite	*OH	65%	10 min	[97]
PFOA (120 μM)	BN/TiO_2_ composite	h_VB_+	98%	2 h (254 nm)	[98]
PFAS	UV Fenton (Fe_3_O_4_ nanoparticles)	ROS	80%	30 min (254 nm)	[99]

## References

[1] Vo H.N.P., Ngo H.H., Guo W., Nguyen T.M.H., Li J., Liang H., Deng L., Chen Z., Nguy T.A.H. (2020). Poly- and perfluoroalkyl substances in water and wastewater: A comprehensive review from sources to remediation. J. Water Process Eng..

[2] Domingo J.L., Nadal M. (2019). Human exposure to per- and polyfluoroalkyl substances (PFAS) through drinking water: A review of the recent scientific literature. Environ. Res..

[3] http://www.pops.int/TheConvention/ThePOPs/tabid/673/Default.aspx.

[4] Teymoorian T., Munoz G., Vo Duy S., Liu J., Sauvé S. (2023). Tracking PFAS in Drinking Water: A Review of Analytical Methods and Worldwide Occurrence Trends in Tap Water and Bottled Water. ACS Environ. Sci. Technol. Water.

[5] Wanninayake D.M. (2021). Comparison of currently available PFAS remediation technologies in water: A review. J. Environ. Manag..

[6] Leung S.C., Shukla P., Chen D., Eftekhari E., An H., Zare F., Ghasemi N., Zhang D., Nguyen N.T., Li Q. (2022). Emerging technologies for PFOS/PFOA degradation and removal: A review. Sci. Total Environ..

[7] Araújo R.J., Rodríguez-Hernandéz J.A., González-González R.B., Macias-Garbett R., Martínez-Ruiz M., Reyes-Pardo H., Martínez S.A., Parra-Arroyo L., Melchor-Martínez E.M., Sosa-Hernández J.E. (2022). Detection and Tertiary Treatment Technologies of Poly-and Perfluoroalkyl Substances in Wastewater Treatment Plants. Front. Environ. Sci..

[8] Ateia M., Alsbaiee A., Karanfil T., Dichtel W. (2019). Efficient PFAS Removal by Amine-Functionalized Sorbents: Critical Review of the Current Literature. Environ. Sci. Technol. Lett..

[9] Park M., Wu S., Lopez I.J., Chang J.Y., Karanfil T., Snyder S.A. (2020). Adsorption of perfluoroalkyl substances (PFAS) in groundwater by granular activated carbons: Roles of hydrophobicity of PFAS and carbon characteristics. Water Res..

[10] Militao I.M., Roddick F.A., Bergamasco R., Fan L. (2021). Removing PFAS from aquatic systems using natural and renewable material-based adsorbents: A review. J. Environ. Chem. Eng..

[11] Yadav S., Ibrar I., Al-Juboori R.A., Singh L., Ganbat N., Kazwini T., Karbassiyazdi E., Samal A.K., Subbiah S., Altaee A. (2022). Updated review on emerging technologies for PFAS contaminated water treatment. Chem. Eng. Res. Des..

[12] Dixit F., Dutta R., Barbeau B., Berube P., Mohseni M. (2021). PFAS removal by ion exchange resins: A review. Chemosphere.

[13] Yang L., He L., Xueb J., Ma Y., Xie Z., Wu L., Huang M., Zhang Z. (2020). Persulfate-based degradation of perfluorooctanoic acid (PFOA) and perfluorooctane sulfonate (PFOS) in aqueous solution: Review on influences, mechanisms and prospective. J. Hazard. Mater..

[14] Huang J., Wang X., Pan Z., Li X., Ling Y., Li L. (2016). Efficient degradation of perfluorooctanoic acid (PFOA) by photocatalytic ozonation. Chem. Eng. J..

[15] Kim J., Xin X., Mamo B.T., Hawkins G.L., Li K., Chen Y., Huang Q., Huang C. (2022). Occurrence and Fate of Ultrashort-Chain and Other Per- and Polyfluoroalkyl Substances (PFAS) in Wastewater Treatment Plants. ACS EST Water.

[16] Manz K.E., Kulaots I., Greenley C.A., Landry P.J., Lakshmi K.V., Woodcock M.J., Hellerich L., Bryant J.D., Apfelbaum M., Pennell K.D. (2023). Low-temperature persulfate activation by powdered activated carbon for simultaneous destruction of perfluorinated carboxylic acids and 1,4-dioxane. J. Hazard. Mater..

[17] Xu T., Zhu Y., Duan J., Xi Y., Tong T., Zhang L., Zhao D. (2020). Enhanced photocatalytic degradation of perfluorooctanoic acid using carbon-modified bismuth phosphate composite: Effectiveness, material synergy and roles of carbon. Chem. Eng. J..

[18] Xia C., Lim X., Yang H., Goodson B.M., Liu J. (2022). Degradation of per- and polyfluoroalkyl substances (PFAS) in wastewater effluents by photocatalysis for water reuse. J. Water Process Eng..

[19] Zhang W., Zhang D., Liang Y. (2019). Nanotechnology in remediation of water contaminated by poly- and perfluoroalkyl substances: A review. Environ. Pollut..

[20] Zhang J., Pang H., Gray S., Ma S., Xie Z., Gao L. (2021). PFAS removal from wastewater by in-situ formed ferric nanoparticles: Solid phase loading and removal efficiency. J. Environ. Chem. Eng..

[21] Birch Q.T., Birch M.E., Nadagouda M.N., Dionysiou D.D. (2022). Nano-enhanced treatment of per-fluorinated and poly- fluorinated alkyl substances (PFAS). Curr. Opin. Chem. Eng..

[22] Yin S., Villagrán D. (2022). Design of nanomaterials for the removal of per- and poly-fluoroalkyl substances (PFAS) in water: Strategies, mechanisms, challenges, and opportunities. Sci. Total Environ..

[23] Duinslaeger N., Radjenovic J. (2022). Electrochemical degradation of per- and polyfluoroalkyl substances (PFAS) using low-cost graphene sponge electrodes. Water Res..

[24] Fang C., Megharaj M., Naidu R. (2017). Electrochemical Advanced Oxidation Processes (EAOP) to degrade per- and polyflluoroalkyl substances (PFASs). J. Adv. Oxid. Technol..

[25] Trojanowicza M., Bojanowska-Czajkaa A., Bartosiewicza I., Kulisa K. (2018). Advanced Oxidation/Reduction Processes treatment for aqueous perfluorooctanoate (PFOA) and perfluorooctanesulfonate (PFOS)–A review of recent advances. Chem. Eng. J..

[26] Saleh N.B., Khalid A., Tian Y., Ayres C., Sabaraya I.V., Pietari J., Hanigan D., Chowdhury I., Apul O.G. (2019). Removal of poly- and per-fluoroalkyl substances from aqueous systems by nano-enabled water treatment strategies. Environ. Sci. Water Res. Technol..

[27] Cui J., Gao P., Deng Y. (2020). Destruction of Per- and Polyfluoroalkyl Substances (PFAS) with Advanced Reduction Processes (ARPs): A Critical Review. Environ. Sci. Technol..

[28] Leonello D., Fendrich M.A., Parrino F., Patel N., Orlandi M., Miotello A. (2021). Light-Induced Advanced Oxidation Processes as PFAS Remediation Methods: A Review. Appl. Sci..

[29] Barisci S., Suri R. (2021). Occurrence and removal of poly/perfluoroalkyl substances (PFAS) in municipal and industrial wastewater treatment plants. Water Sci. Technol..

[30] Venkatesan A.K., Lee C.S., Gobler C.J. (2022). Hydroxyl-radical based advanced oxidation processes can increase perfluoroalkyl substances beyond drinking water standards: Results from a pilot study. Sci. Total Environ..

[31] Alalm M.G., Boffito D.C. (2022). Mechanisms and pathways of PFAS degradation by advanced oxidation and reduction processes: A critical review. Chem. Eng. J..

[32] Ambaye T.G., Vaccari M., Prasad S., Rtimi S. (2022). Recent progress and challenges on the removal of per- and poly-fluoroalkyl substances (PFAS) from contaminated soil and water. Environ. Sci. Pollut. Res..

[33] Eun H., Shimamura K., Asano T., Yamazaki E., Taniyasu S., Yamashita N. (2022). Removal of perfluoroalkyl substances from water by activated carbons: Adsorption of perfluorooctane sulfonate and perfluorooctanoic acid. Environ. Monit. Contam. Res..

[34] Riegel M., Haist-Gulde B., Sacher F. (2023). Sorptive removal of short-chain perfluoroalkyl substances (PFAS) during drinking water treatment using activated carbon and anion exchanger. Environ. Sci. Eur..

[35] Zango Z.U., Khoo K.S., Garba A., Kadir H.A., Usman F., Zango M.U., Oh W.D., Lim J.W. (2023). A review on superior advanced oxidation and photocatalytic degradation techniques for perfluorooctanoic acid (PFOA) elimination from wastewater. Environ. Res..

[36] Vellanki1 B.P., Batchelor B., Abdel-Wahab A. (2013). Advanced Reduction Processes: A New Class of Treatment Processes. Environ. Eng. Sci..

[37] Gu Y., Liu T., Zhang Q., Dong W. (2017). Efficient decomposition of perfluorooctanoic acid by a high photon flux UV/sulfite process: Kinetics and associated toxicity. Chem. Eng. J..

[38] Ren Z., Bergmann U., Leiviska T. (2021). Reductive degradation of perfluorooctanoic acid in complex water matrices by using the UV/sulfite process. Water Res..

[39] Cheng J.H., Liang X.Y., Yang S.W., Hu Y.Y. (2014). Photochemical defluorination of aqueous perfluorooctanoic acid (PFOA) by VUV/Fe^3+^ system. Chem. Eng. J..

[40] Gu Y., Liu T., Wang H., Han H., Dong W. (2017). Hydrated electron based decomposition of perfluorooctane sulfonate (PFOS) in the VUV/sulfite system. Sci. Total Environ..

[41] Cardoso I.M.F., Cardoso R.M.F., Esteves da Silva J.C.G. (2021). Advanced Oxidation Processes Coupled with Nanomaterials for Water Treatment. Nanomaterials.

[42] Cardoso R.M.F., Cardoso I.M.F., da Silva L.P., Esteves da Silva J.C.G. (2022). Copper(II)-Doped Carbon Dots as Catalyst for Ozone Degradation of Textile Dyes. Nanomaterials.

[43] Cardoso I.M.F., Cardoso R.M.F., Pinto da Silva L., Esteves da Silva J.C.G. (2022). UV-Based Advanced Oxidation Processes of Remazol Brilliant Blue R Dye Catalyzed by Carbon Dots. Nanomaterials.

[44] Lin A.Y.C., Panchangam S.C., Chang C.Y., Hong P.K.A., Hsueh H.F. (2012). Removal of perfluorooctanoic acid and perfluorooctane sulfonate via ozonation under alkaline condition. J. Hazard. Mater..

[45] Schroder H.F., Meesters R.J.W. (2005). Stability of fluorinated surfactants in advanced oxidation processes—A follow up of degradation products using flow injection–mass spectrometry, liquid chromatography–mass spectrometry and liquid chromatography–multiple stage mass spectrometry. J. Chromatogr. A.

[46] Franke V., Schäfers M.D., Lindberg J.J., Ahrens L. (2019). Removal of per- and polyfluoroalkyl substances (PFASs) from tap water using heterogeneously catalyzed ozonation. Environ. Sci. Water Res. Technol..

[47] Dai X., Xie Z., Dorian B., Gray S., Zhang J. (2019). Comparative study of PFAS treatment by UV, UV/ozone, and fractionations with air and ozonated air. Environ. Sci. Water Res. Technol..

[48] Merényi G., Lind J., Naumov S., Sonntag C.V. (2010). Reaction of Ozone with Hydrogen Peroxide (Peroxone Process): A Revision of Current Mechanistic Concepts Based on Thermokinetic and Quantum-Chemical Considerations. Environ. Sci. Technol..

[49] Rekhate C.V., Srivastava J.K. (2020). Recent advances in ozone-based advanced oxidation processes for treatment of wastewater- A review. Chem. Eng. J. Adv..

[50] Sauleda R., Brillas E. (2001). Mineralization of aniline and 4-chlorophenol in acidic solution by ozonation catalyzed with Fe^2+^ and UVA light. Appl. Catal. B Environ..

[51] Vatankhah H., Bahareh Tajdini B., Milstead R.P., Clevenger E., Murray C., Knappe D., Remucal C.K., Bellona C. (2022). Impact of ozone-biologically active filtration on the breakthrough of Perfluoroalkyl acids during granular activated carbon treatment of municipal wastewater effluent. Water Res..

[52] Javed H., Lyu C., Sun R., Zhang D., Alvarez P.J.J. (2020). Discerning the inefficacy of hydroxyl radicals during perfluorooctanoic acid degradation. Chemosphere.

[53] Giria R.R., Ozaki H., Okada T., Taniguchi S., Takanami R. (2012). Factors influencing UV photodecomposition of perfluorooctanoic acid in water. Chem. Eng. J..

[54] Umar M. (2021). Reductive and Oxidative UV Degradation of PFAS—Status, Needs and Future Perspectives. Water.

[55] Xu B., Ahmed M.B., Zhou J.L., Ali Altaee A. (2020). Visible and UV photocatalysis of aqueous perfluorooctanoic acid by TiO_2_ and peroxymonosulfate: Process kinetics and mechanistic insights. Chemosphere.

[56] Hori H., Hayakawa E., Einaga H., Kutsuna S., Koike K., Ibusuki T., Kiatagawa H., Arakawa R. (2004). Decomposition of Environmentally Persistent Perfluorooctanoic Acid in Water by Photochemical Approaches. Environ. Sci. Technol..

[57] Hori H., Yamamoto A., Hayakawa E., Taniyasu S., Yamashita N., Kutsuna S., Kiatagawa H., Arakawa R. (2005). Efficient Decomposition of Environmentally Persistent Perfluorocarboxylic Acids by Use of Persulfate as a Photochemical Oxidant. Environ. Sci. Technol..

[58] Hori H., Murayama M., Inoue N., Ishida K., Kutsuna S. (2010). Efficient mineralization of hydroperfluorocarboxylic acids with persulfate in hot water. Catal. Today.

[59] Liu C.S., Higgins C.P., Wang F., Shih K. (2012). Effect of temperature on oxidative transformation of perfluorooctanoic acid (PFOA) by persulfate activation in water. Sep. Purif. Technol..

[60] Yang S., Cheng J., Sun J., Hu Y., Liang X. (2013). Defluorination of Aqueous Perfluorooctanesulfonate by Activated Persulfate Oxidation. PLoS ONE.

[61] Park S., Lee L.S., Medina V.F., Zull A., Waisner S. (2016). Heat-activated persulfate oxidation of PFOA, 6:2 fluorotelomer sulfonate, and PFOS under conditions suitable for in-situ groundwater remediation. Chemosphere.

[62] Gatto S., Sansotera M., Persico F., Gola M., Pirola C., Panzeri W., Navarrini W., Bianchi C.L. (2015). Surface fluorination on TiO_2_ catalyst induced by photodegradation of perfluorooctanoic acid. Catal. Today.

[63] Chen M.J., Lo S.L., Lee Y.C., Huang C.C. (2015). Photocatalytic decomposition of perfluorooctanoic acid by transition-metal modified titanium dioxide. J. Hazard. Mater..

[64] Li F., Wei Z., He K., Blaney L., Cheng X., Xu T., Liud W., Zhao D. (2020). A concentrate-and-destroy technique for degradation of perfluorooctanoic acid in water using a new adsorptive photocatalyst. Water Res..

[65] Wang J., Cao C., Wang Y., Wang Y., Sun B., Zhu L. (2020). In situ preparation of *p-n* BiOI@Bi_5_O_7_I heterojunction for enhanced PFOA photocatalytic degradation under simulated solar light irradiation. Chem. Eng. J..

[66] Yang Y., Ji W., Li X., Zheng Z., Bi F., Yang M., Xu J., Zhang X. (2021). Insights into the degradation mechanism of perfluorooctanoic acid under visible-light irradiation through fabricating flower-shaped Bi_5_O_7_I/ZnO *n-n* heterojunction microspheres. Chem. Eng. J..

[67] Kurian M. (2021). Advanced oxidation processes and nanomaterials—A review. Clean. Eng. Technol..

[68] Arora B., Attri P. (2020). Carbon Nanotubes (CNTs): A Potential Nanomaterial for Water Purification. J. Compos. Sci..

[69] Steven J., Chow S.J., Croll H.C., Ojeda N., Klamerus J., Capelle R., Oppenheimer J., Jacangelo J.G., Schwab K.J., Prasse C. (2022). Comparative investigation of PFAS adsorption onto activated carbon and anion exchange resins during long-term operation of a pilot treatment plant. Water Res..

[70] Das R., Leo B.F., Murphy F. (2018). The Toxic Truth About Carbon Nanotubes in Water Purification: A Perspective View. Nanoscale Res. Lett..

[71] Esteves da Silva J.C.G., Gonçalves H. (2011). Analytical and bioanalytical applications of carbon dots. TrAC Trends Anal. Chem..

[72] Meng W., Bai X., Wang B., Liu Z., Lu S., Yang B. (2019). Biomass-Derived Carbon Dots and Their Applications. Energy Environ. Mater..

[73] Manzoor S., Dar A.H., Dash K.D., Pandey V.K., Srivastava S., Bashir I., Khan S.A. (2023). Carbon dots applications for development of sustainable technologies for food safety: A comprehensive review. Appl. Food Res..

[74] Wang J., Jiang J., Li F., Zou J., Xiang K., Wang H., Li Y., Li X. (2023). Emerging carbon-based quantum dots for sustainable photocatalysis. Green Chem..

[75] Chen P.Y., Wang B., Chen W.G., Zhuang S., Chen Y., Liu H.J. (2022). Carbon-dot-modified polyacrylonitrile fiber as recyclable adsorbent for removing anionic, cationic, and zwitterionic perfluorooctane sulfonates from water. Colloids Surf. A Physicochem. Eng. Asp..

[76] Wang W.R., Chen P.Y., Deng J., Chen Y., Liu H.J. (2022). Carbon-dot hydrogels as superior carbonaceous adsorbents for removing perfluorooctane sulfonate from water. Chem. Eng. J..

[77] Chen Q., Zhu P., Xiong J., Gao L., Tan K. (2019). A sensitive and selective triple-channel optical assay based on red-emissive carbon dots for the determination of PFOS. Microchem. J..

[78] Rodriguez K.L., Hwang J.-H., Esfahani A.R., Sadmani A.H.M.A., Lee W.H. (2020). Recent Developments of PFAS-Detecting Sensors and Future Direction: A Review. Micromachines.

[79] Wang Y., Darling S.B., Chen J. (2021). Selectivity of Per- and Polyfluoroalkyl Substance Sensors and Sorbents in Water. ACS Appl. Mater. Interfaces.

[80] Li J., Li X., Da Y., Yu J., Long B., Zhang P., Bakker C., McCarl B.A., Yuan J.S., Dai S.D. (2022). Sustainable environmental remediation via biomimetic multifunctional lignocellulosic nano-framework. Nat. Commun..

[81] Silva A.R., Duarte M.S., Alves M.M., Pereira L. (2022). Bioremediation of Perfluoroalkyl Substances (PFAS) by Anaerobic Digestion: Effect of PFAS on Different Trophic Groups and Methane Production Accelerated by Carbon Materials. Molecules.

[82] Douna B.K., Yousefi H. (2023). Removal of PFAS by Biological Methods. Asian Pac. J. Environ. Cancer.

[83] Smith S.J., Wiberg K., McCleaf P., Ahrens L. (2022). Pilot-Scale Continuous Foam Fractionation for the Removal of Per- and Polyfluoroalkyl Substances (PFAS) from Landfill Leachate. ACS EST Water.

[84] Wang Y., Ji Y., Tishchenko V., Huang Q. (2023). Removing per- and polyfluoroalkyl substances (PFAS) in water by foam fractionation. Chemosphere.

[85] Morrison A.L., Strezov V., Niven R.K., Taylor M.P., Wilson S.P., Wang J., Burns D.J., Murphy P.J.C. (2023). Impact of Salinity and Temperature on Removal of PFAS Species from Water by Aeration in the Absence of Additional Surfactants: A Novel Application of Green Chemistry Using Adsorptive Bubble Fractionation. Ind. Eng. Chem. Res..

[86] Jia L., Liu W., Cao J., Wu Z., Yang C. (2021). Recovery of nanoparticles from wastewater by foam fractionation: Regulating bubble size distribution for strengthening foam drainage. J. Environ. Chem. Eng..

[87] Lei X., Lian Q., Zhang X., Karsili T.K., Holmes W., Chen Y., Zappi M.E., Gang D.D. (2023). A review of PFAS adsorption from aqueous solutions: Current approaches, engineering applications, challenges, and opportunities. Environ. Pollut..

[88] Yin S., López J.F., Solís J.J.C., Wong M.S., Villagrán D. (2023). Enhanced adsorption of PFOA with nano MgAl_2_O_4_@CNTs: Influence of pH and dosage, and environmental conditions. J. Hazard. Mater. Adv..

[89] Karbassiyazdi E., Medha Kasula M., Sweta Modak S., Pala J., Kalantari M., Altaee A., Esfahani M.R., Razmjou A. (2023). A juxtaposed review on adsorptive removal of PFAS by metal-organic frameworks (MOFs) with carbon-based materials, ion exchange resins, and polymer adsorbents. Chemosphere.

[90] Wen Y., Gómez A.R., Day G.S., Smith M.F., Yan T.H., Ozdemir R.O.K., Gutierrez O., Sharma V.K., Ma X., Zhou H.C. (2022). Integrated Photocatalytic Reduction and Oxidation of Perfluorooctanoic Acid by Metal−Organic Frameworks: Key Insights into the Degradation Mechanisms. J. Am. Chem. Soc..

[91] Chen C., Ma Q., Liu F., Gao J., Li X., Sun S., Hong Yao H., Liu C., Young J., Zhan W. (2021). Photocatalytically reductive defluorination of perfluorooctanoic acid (PFOA) using Pt/La_2_Ti_2_O_7_ nanoplates: Experimental and DFT assessment. J. Hazard. Mater..

[92] Wang J., Cao C., Zhang Y., Zhang Y., Zhu L. (2021). Underneath mechanisms into the super effective degradation of PFOA by BiOF nanosheets with tunable oxygen vacancies on exposed (101) facets. Appl. Catal. B Environ..

[93] Ji H., Ni J., Zhao D., Liu W. (2022). Application of Titanate Nanotubes for Photocatalytic Decontamination in Water: Challenges and Prospects. ACS EST Eng..

[94] Zhu Y., Xu T., Zhao D., Li F., Liu W., Wang B., An B. (2021). Adsorption and solid-phase photocatalytic degradation of perfluorooctane sulfonate in water using gallium-doped carbon-modified titanate nanotubes. Chem. Eng. J..

[95] Zhu Y., Xu T., Zhao D. (2022). Metal-doped carbon-supported/modified titanate nanotubes for perfluorooctane sulfonate degradation in water: Effects of preparation conditions, mechanisms, and parameter optimization. Sci. Total Environ..

[96] Zhu Y., Ji H., He K., Blaney L., Xu T., Zhao D. (2022). Photocatalytic degradation of GenX in water using a new adsorptive photocatalyst. Water Res..

[97] Kundu S., Radian A. (2022). Surface confinement of per-fluoroalkyl substances on an iron-decorated clay-cyclodextrin composite enables rapid oxidation by hydroxyl radicals. Chem. Eng. J..

[98] Duan L., Wang B., Kimberly Heck N., Clark C.A., Wei J., Wang M., Metz J., Wu G., Tsai A.L.T., Guo S. (2022). Titanium oxide improves boron nitride photocatalytic degradation of perfluorooctanoic acid. Chem. Eng. J..

[99] Schlesinger D.R., McDermott C., Le N.Q., Ko J.S., Johnson J.K., Demirev P.A., Xia Z. (2022). Destruction of per/poly-fluorinated alkyl substances by magnetite nanoparticle-catalyzed UV-Fenton reaction. Environ. Sci. Water Res. Technol..

[100] Metz J., Zuo P., Wang B., Wong M.S., Alvarez P.J.J. (2022). Perfluorooctanoic acid Degradation by UV/Chlorine. Environ. Sci. Technol. Lett..

[101] Trang B., Li Y., Xue X.S., Ateia M., Houk K.N., Dichtel W.R. (2022). Low-temperature mineralization of perfluorocarboxylic acids. Science.

